# Exosomes taken up by neurons hijack the endosomal pathway to spread to interconnected neurons

**DOI:** 10.1186/s40478-018-0514-4

**Published:** 2018-02-15

**Authors:** Juan Carlos Polanco, Chuanzhou Li, Nela Durisic, Robert Sullivan, Jürgen Götz

**Affiliations:** 1 Clem Jones Centre for Ageing Dementia Research (CJCADR), Queensland Brain Institute (QBI), The University of Queensland, Brisbane, QLD 4072 Australia; 2 Queensland Brain Institute (QBI), The University of Queensland, Brisbane, QLD 4072 Australia

**Keywords:** Alzheimer, Tau, Spreading, Exosomes, Endosomes, Protein aggregates, Organelle fusion, Axonal transport

## Abstract

In Alzheimer disease and related disorders, the microtubule-associated protein tau aggregates and forms cytoplasmic lesions that impair neuronal physiology at many levels. In addition to affecting the host neuron, tau aggregates also spread to neighboring, recipient cells where the misfolded tau aggregates, in a manner similar to prions, actively corrupt the proper folding of soluble tau, and thereby impair cellular functions. One vehicle for the transmission of tau aggregates are secretory nanovesicles known as exosomes. Here, we established a simple model of a neuronal circuit using a microfluidics culture system in which hippocampal neurons A and B were seeded into chambers 1 and 2, respectively, extending axons via microgrooves in both directions and thereby interconnecting. This system served to establish two models to track exosome spreading. In the first model, we labeled the exosomal membrane by coupling tetraspanin CD9 with either a green or red fluorescent tag. This allowed us to reveal that interconnected neurons exchange exosomes only when their axons extend in close proximity. In the second model, we added exosomes isolated from the brains of tau transgenic rTg4510 mice (i.e. exogenous, neuron A-derived) to neurons in chamber 1 (neuron B) interconnected with neuron C in chamber 2. This allowed us to demonstrate that a substantial fraction of the exogenous exosomes were internalized by neuron B and passed then on to neuron C. This transportation from neuron B to C was achieved by a mechanism that is consistent with the hijacking of secretory endosomes by the exogenous exosomes, as revealed by confocal, super-resolution and electron microscopy. Together, these findings suggest that fusion events involving the endogenous endosomal secretory machinery increase the pathogenic potential and the radius of action of pathogenic cargoes carried by exogenous exosomes.

**Electronic supplementary material:**

The online version of this article (10.1186/s40478-018-0514-4) contains supplementary material, which is available to authorized users.

## Introduction

Alzheimer disease (AD), the most common form of aging dementia, is characterized by problems with memory, thinking and behavior [50]. These clinical features are strongly associated with the accumulation of two types of insoluble protein deposits in the AD brain, which are composed of either the amyloid-β (Aβ) peptide or the microtubule-associated protein tau and impair neuronal function at many levels [5, 32, 44, 50]. The Aβ deposits are referred to as amyloid plaques and are found in the interstitial space of the brain, whereas the lesions composed of aggregated tau, known as neurofibrillary tangles (NFTs), are intraneuronal [5, 32, 44, 50]. Tau pathology progresses through well-defined stereotyped stages, which appears to be initiated in the locus coeruleus and slowly spreads via the entorhinal cortex and hippocampus to the neocortex [12, 13]; however the role of the locus coeruleus is controversial [4]. This pattern of tau spreading led to the suggestion that AD progression occurs by neuron-to-neuron transmission involving trans-synaptic transport of seeds of tau aggregation from affected to anatomically interconnected recipient neurons [12, 13]. It has since been established that the intercellular transfer of misfolded forms of tau known as “seeds” contributes to the progression of AD, with tau seeds acting in a manner similar to prions, triggering the robust conversion of soluble tau into insoluble large filamentous aggregates and NFTs [14, 30, 50].

Several modes of neuron-to-neuron transfer of tau seeds have been described, including via extracellular vesicles such as exosomes [22, 51, 66], trans-synaptically mediated transfer of tau aggregates between interconnected neurons [15, 23], tunneling nanotubes [61] or the uptake of free-floating tau aggregates and fibrils [30, 35]. In vitro evidence suggests that reducing the pool of extracellular tau seeds, irrespective of whether these are moving freely or are transported by exosomes or any other mechanism of inter-neuronal transfer, results in an in vivo reduction of tau pathology by maintaining the level of extracellular tau seeds below a pathological concentration threshold [5, 15, 29, 30, 36, 51, 61]. Our research focuses on exosomes, membranous secreted nanovesicles 30–150 nm in size, that are produced in late endosomes by the inward budding of the endosomal membrane, which is progressively pinched off to generate and accumulate intraluminal nanovesicles [11, 38, 45]. The late endosome, loaded with intraluminal nanovesicles, then gradually develops into large multivesicular bodies (MVBs). These MVBs can fuse with the plasma membrane to release the intraluminal nanovesicles into the extracellular environment, and once secreted these free nanovesicles are termed “exosomes” [11, 38, 45].

A number of studies have shown that exosomes can transport Aβ and derivatives of the amyloid precursor protein (APP) from which Aβ originates [48, 52, 58]. They also contain phosphorylated tau as demonstrated for exosomes that have been isolated from the blood and cerebrospinal fluid of AD patients [26, 55]. Furthermore, immuno-electron microscopy of AD brain tissue has revealed that human Aβ plaques are enriched in exosomal proteins [52]. Mouse models of AD have been instrumental in demonstrating that exosome reduction in vivo is associated with a lower Aβ plaque load in the brain [20, 21]. Similarly, depletion of microglia and inhibition of exosome synthesis has been found to halt tau propagation in the brains of tauopathy mouse models [3]. Taken together, these studies support the notion that reducing exosome secretion results in reduced Aβ plaque formation and tau propagation. Related to this, we have demonstrated that tau seeds are contained within exosomes isolated from the brains of tauopathy mice, that they have a distinct phosphorylation pattern, and that only exosomes derived from cells undergoing tau aggregation are able to seed and corrupt soluble tau in recipient cells, a phenomenon that occurs in a threshold-dependent manner [6, 51].

An important question in the field is how the seeds are taken up and handled by recipient cells. Here, neuron-to-neuron transmission of exosomes emerges as an important pathomechanism for the progression of AD. Such a mechanism implies that a neuron generates exosomes in endosomes, an organelle which is more abundant in the soma than in axons [65], after which the mature MVBs undergo anterograde transport along the axons until they fuse with the plasma membrane to release the exosome at the synapse of an interconnected cell. Evidence for such a trans-synaptic mechanism has been provided by studies in *Drosophila* which investigated exosomes carrying Wnt signals at the neuromuscular junction [41, 42]. In our study, we used simple microfluidics circuit systems to demonstrate that exosomes are not only being exchanged between interconnected neurons A and B, but that a recipient neuron C can receive exosomes that have either been generated by an interconnected neuron B or are passed on via this interconnected neuron after processing of ‘exogenous’ exosomes that have been internalized from neuron A. This ‘longer-distance action’ of exosomes appears to be linked to the hijacking of secretory endosomes present in neuron B of this simple circuit. We discuss how such fusion events potentially increase the pathogenic potential and the radius of action of pathogenic cargoes carried by exogenous exosomes.

## Materials and methods

### Mouse strains and collection of brain tissue

C57BL/6 mice were used at embryonic day 17 (E17) to isolate hippocampal neurons for tissue culture experiments. rTg4510 mice expressing human four-repeat tau with the P301L mutation linked to hereditary tauopathy [56] were used at 4–6 months of age for exosome isolation. Animal experimentation was approved by the Animal Ethics Committee of the University of Queensland (approval number QBI/412/14/NHMRC).

### Isolation and purification of brain exosomes

Exosomes were isolated from the interstitial space of mouse brains using a previously established protocol [48, 51]. In brief, each brain was dissected and gently chopped before being incubated in 7 ml of 0.2% *w*/*v* Collagenase type III (LS004182, Worthington) in serum-free Hibernate-A medium (A12475–01, Life Technologies) for 30 min at 37 °C. The dissociation reaction was stopped with 14 ml of ice-cold Hibernate-A containing 1× Complete protease inhibitor cocktail (Roche), 50 mM NaF and 200 nM Na_3_VO_4_. The tissue was then gently dissociated with a 10 ml pipette, keeping the cells intact during pipetting them up and down, followed by a series of differential 4 °C centrifugations at 300 g for 10 min, 2000 g for 10 min and 10,000 g for 30 min to sequentially discard the pellet containing cells, membranes, and nanodebris, respectively. The supernatant from the final centrifugation step was passed through a 0.22 μm syringe filter (Millex-GP, Millipore) and centrifuged at 120,000 g for 70 min at 4 °C to pellet the exosomes. The pellet containing the exosomes was then washed with 5 ml phosphate-buffered saline (PBS, 17-516Q, Lonza), after which the pellets from five mouse brains per genotype (25 ml) were pooled. This preparation was centrifuged at 120,000 g for 70 min at 4 °C to obtain a pellet that was resuspended in 2 ml of 0.95 M sucrose in 20 mM HEPES (15630–080, Life Technologies), then purified using a sucrose step gradient column (six 2 ml steps at 2.0, 1.65, 1.3, 0.95, 0.6 and 0.25 M sucrose from bottom to top). The sucrose gradient was centrifuged at 200,000 g for 16 h at 4 °C. The exosome-containing fraction 3 (0.95 M; ρ = 1.12 g/ml sucrose) was collected together with the interphase and resuspended in 6 ml ice-cold PBS, followed by a 120,000 g centrifugation for 70 min at 4 °C. Finally, the sucrose-purified exosome pellet was resuspended in 100 μl PBS containing 1× Complete protease inhibitor cocktail (Roche). A 10 μl aliquot of exosomes in PBS was homogenized with 10 μl of 2× RIPA buffer (300 mM NaCl, 100 mM Tris-HCl pH 7.4, 0.50% (*w*/*v*) sodium deoxycholate, 0.2% (*v*/v) Nonidet P-40) to determine the protein content with a Micro BCA™ Protein Assay Kit (23,235, Thermo-Fisher).

### Fluorescence labeling of exosome membranes

To track exogenous exosomes isolated from mouse brains, we labeled their membranes with an appropriate fluorescent stain that stably incorporates a fluorescent dye with long aliphatic tails into the exosome membrane. In our study, the fluorescent membrane probes CellVue® Claret Far-Red Fluorescent Membrane Linker (Sigma), PKH67 Green Fluorescent Membrane Linker (Sigma) and FM™ 1–43FX Fixable Membrane Stain (Thermo-Fisher) were used to separately label sucrose-purified exosomes according to the manufacturer’s instructions. The labeling reaction was stopped with 6 ml of 2% bovine serum albumin, followed by ultra-centrifugation at 100,000 g for 70 min, washing with PBS and another round of ultra-centrifugation, followed by resuspension of the fluorescently labeled exosomes in PBS.

### Primary neuronal culture and microfluidic devices

Hippocampal neurons were isolated by standard methods using C57BL/6 mice sacrificed at E17 and grown in culture chamber microfluidic devices (Xona Microfluidics) placed on 24 × 60 mm coverslips (#1,5 Menzel-Glaser) that had been coated with poly-D-lysine (PDL), to form a non-plasma bond with the device. 60,000–80,000 neurons were plated per chamber using Neurobasal medium (21,103,049, Thermo-Fisher) supplemented with 5% fetal bovine serum (FBS; Hyclone), 2% B27 (17,504,044, Thermo-Fisher), 1 mM GlutaMAX (35,050,061, Thermo-Fisher) and 50 U/ml penicillin/streptomycin (15,070,063, Thermo-Fisher). The medium was changed to serum-free Neurobasal medium minus phenol red (12,348,017, Thermo-Fisher), supplemented with 28 nM 2-mercaptoethanol (21,985,023, Thermo-Fisher) 24 h post-seeding, and half of the medium was changed twice a week. A hydrostatic pressure gradient that prevents diffusion between culture chamber (Ch) 1 and Ch2 was established by adding twice the volume of culture medium to Ch2. To perform electron microscopy, the microfluidic devices were placed on plastic culture dishes coated with PDL. All cultures were maintained at 37 °C and 5% CO_2_ for up to 12 days. Neurons grown in the microfluidic devices were analyzed at 8–12 days in vitro (DIV8–12).

### Plasmids, virus preparation and electroporations

CD9 is a tetraspanin that is expressed on plasma, endosomal and exosomal membranes [2, 8, 64]. To label and track exosomes, dispersed primary hippocampal neurons were transfected with the plasmids mCherry-CD9–10 (Addgene # 55013) and Dendra2-CD9–10 (Addgene #57705), kind gifts from Dr. Michael Davidson to Addgene. For transfection, 4 × 10^6^ neurons were electroporated with 4 μg plasmid DNA using a Nucleofector™ 2b device and the Amaxa Basic Primary Neurons Nucleofector® Kit (VPI-1003, Lonza). After electroporation, the neurons were resuspended in FBS-containing Neurobasal plating medium, centrifuged for 5 min at 100 g and then resuspended in Neurobasal plating medium to obtain a concentration of 8000 neurons per μl before seeding the neurons in the chambers of the microfluidic device. To detect endosomes, neurons were also transduced with a commercial baculovirus expressing the late endosomal marker LAMP1 (lysosomal-associated membrane protein 1) tagged with RFP (Thermo, C10597). After 48 h they were fixed in paraformaldehyde and imaged.

### Confocal microscopy and image analysis

Fluorescence images at a 63× magnification were obtained with a Zeiss LSM 710 inverted laser scanning confocal microscope using a 1-2× optical zoom. For fluorescent particle quantification of endosomes, 4–10 non-overlapping confocal images at a 63× magnification were analyzed per sample using the open source ImageJ software (version 1.51r, Wayne Rasband, National Institutes of Health, Bethesda). The acquisition parameters remained invariable for all images. The fluorescence signal was adjusted by image segmentation applying a ‘Triangle’ threshold to specifically detect endosomal fluorescent particles in the green (Dendra2-CD9) and red (mCherry-CD9) channels. The thresholded binary images with particles were processed to reduce noise using the Image-J ‘Despeckle’ plugin, followed by the ‘Watershed’ filter to separate overlapping particles in the binary images. Particles were quantified in the segmented images with the ‘Analyze Particles’ ImageJ plugin using the parameter size 0.2–10 squared microns (μm [2]) and circularity 0.1–1 (value of 1 indicating a perfect circle). Occasionally, fluorescent detection of CD9 in the plasma membrane generated fragments of neurites that were included as endosomal particles by ImageJ. Those inconsistencies were manually curated and excluded from the analysis using the ROI Manager. Then, the ‘Image Calculator’ plugin was used to multiply the segmented binary images from the green and red channels in order to quantify the number of endosomal particles that had both colors.

### Super-resolution microscopy

Super-resolution microscopy was performed as a combination of Photo-Activated Localization Microscopy (PALM) using the fluorescent protein Dendra2 as the label for CD9 and direct Stochastic Optical Reconstruction Microscopy (dSTORM) using CellVue Claret. All super-resolution experiments were performed on an Elyra STORM/SIM microscope (Carl Zeiss, GmbH) equipped with a 100× oil-immersion objective, a focus lock system, an EMCCD Andor iXon Ultra 897 camera (Andor Technologies) and a super-resolution multiband dichroic and emission filter set (405/488/561/635-A-000,m Semrock). Neurons were imaged in highly inclined illumination mode at 20 kHz. Zen 2012 SP2 (black) software (Carl Zeiss, GmbH) was used for image reconstruction and channel alignment in the dual color experiments.

### Electron microscopy and DAB photoconversion

Exosomes labeled with FM™1–43FX Fixable Membrane Stain (Thermo-Fisher) were added to primary hippocampal neurons grown in chamber 1 of microfluidic devices bound to 6 cm plastic culture dishes (Falcon, 353,002). 24 h after exosome uptake, the cultures were briefly washed 3× for 5 min with PBS to remove cellular debris and non-internalized exosomes, fixed with 4% paraformaldehyde (Sigma, 15,827) in PBS for 20 min, and again washed in PBS 3× for 5 min. The cultures were then incubated in 100 mM ammonium chloride solution (Sigma, 213,330) for 20 min, after which they were washed in PBS. Fixed cultures were incubated in 1.5 mg/ml diaminobenzidine (DAB, Sigma D5637) in PBS for 30 min at 4 °C. DAB forms a stable, insoluble precipitate that has a dark appearance and can be easily distinguished by electron microscopy [33, 34]. We therefore photoconverted the FM™ 1–43FX stain into a DAB precipitate using a light box equipped with four 24-watt lamps (Atiaquaristik), two lamps at 420–460 nm and two at 400–500 nm, refrigerated with a cooling fan to prevent heating of the samples. Fluorescence bleaching and complete DAB photoconversion were confirmed by direct visualization using an axioscope (Zeiss).

For electron microscopy, samples were processed using protocols of the Biowave Pro+ (Pelco) microwave tissue processor maintaining the samples at room temperature. Biowave procedures included fixation in 2.5% glutaraldehyde (Proscitech, C003) in PBS for 3 min, osmication with 4% osmium (Proscitech, C010) and 3% potassium ferrocyanide (Sigma, P8131), and washing in dH_2_O 5× for 3 min, followed by incubation in 1% thiocarbohydrazide (Sigma, 88,535) for 20 min. After this, the samples were stained in 2% osmium tetroxide, washed in dH_2_O 5 × for 3 min and then stained in 1% uranyl acetate (Proscitech, C079) for 30 min. After 5 washes in dH_2_O for 3 min, the samples were stained in 20 mM lead aspartate (Proscitech, C151, Sigma, A9256) for 30 min at 60°C. Cultures were dehydrated through a graded series of ethanol followed by 100% acetone and then infiltrated into Epon resin (C038), followed by resin polymerization at 60 °C for 48 h. Resin-embedded samples were cut at 70 nm using a diamond knife (Diatome) and then collected onto 200 × mesh copper grids (Proscitech, GCu200) and imaged on a Hitachi HT7700 transmission electron microscope at 80 kV using an AXT 2Kx2K CMOS digital camera.

## Results

### Establishing neuronal circuits with microfluidic devices

**Fig. 1:**
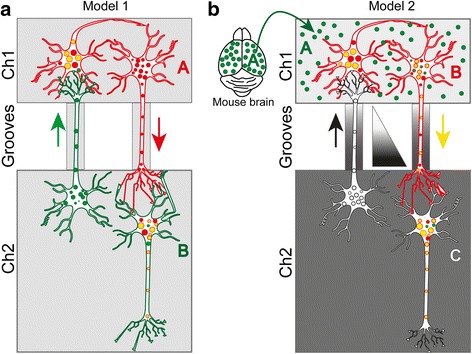
Schematic of experimental designs used to establish neuronal circuits using microfluidic devices. Neurons expressing the tetraspanin CD9 together with either a red or green fluorescent tag were used to track exosomal, endosomal and plasma membranes. **a** Model 1 of two differently colored neurons (A-red and B-green) seeded separately into the two chambers Ch1 and Ch2. Red axons extend from Ch1 to Ch2, but green axons can also project from Ch2 to Ch1. The potential combination of colors by transmission of exosomes is shown in yellow. **b** Model 2 of three interconnected neurons (A-green, B-red, C-no color) seeded into chambers Ch1 and Ch2. The exosomes from A (green) are internalized by B (red) and can potentially be anterogradely transported to C (no color). Combination of colors is shown in yellow. The gradient of the triangle depicts a hydrostatic pressure gradient that prevents diffusion from Ch1 to Ch2. Note that sizes of neurons and culture chambers are not shown at scale

We tested different neuronal cultures in microfluidic devices in order to identify an appropriate neuronal circuit to analyze exosome spreading and tracking. Neuronal cultures grown in the chambers of microfluidic devices are separated by thin microgrooves, which allows for fluidically isolating neuronal cell bodies (somata) from axons (but not dendrites), with axons extending into a connected chamber and thereby establishing synaptic connections [15, 16, 23, 62]. We first tested triple chamber devices (Ch1, Ch2, Ch3) seeded with neurons A, B and C, respectively, interconnected with microgrooves for axonal projections. However, we found that from 8 to 9 days in vitro (DIV8–9) onwards, axons from red neurons seeded in Ch1 grew into Ch3, thereby bypassing neuron B and establishing direct connections A-C, confounding our experimental design (Additional file 1: Figure S1). We therefore opted for the two models that are illustrated in Fig. 1.

(i) Model 1 (Fig. 1a) connects two neurons (A and B) that were individually labeled using a discriminating fluorescence marker (red or green). CD9 was used as a marker for plasma, endosomal and exosomal membranes. To label and track exosomes, dispersed primary hippocampal neurons were transfected with either mCherry-CD9–10 (red) or Dendra2-CD9–10 (green) in chambers Ch1 and Ch2, respectively. The exchange of fluorescence in either direction can be detected with model system 1.
(ii) Model 2 (Fig. 1b) is a proxy for the connection of three neurons (A, B and C) exchanging exosomes and separated in Ch1 and Ch2. Instead of having an intact neuron A, we used brain-derived exosomes from rTg4510 mice [51, 56] labeled with a green fluorescent membrane dye (PKH67) that were added to neuron B (red, expressing mCherry-CD9) in Ch1, whereas the interconnected neuron C in Ch2 was a ‘non-fluorescent’ neuron. Diffusion from Ch1 to Ch2 of brain neuron A-derived exosomes was prevented by establishing a hydrostatic pressure gradient by adding twice the volume of culture medium to Ch2 (Fig. 1b).

### Interconnected neurons exchange exosomes

Exosomes are highly enriched in the protein superfamily termed tetraspanins, which organize plasma membrane microdomains [2]. The tetraspanins CD9, CD63, CD81 and CD151 have a broad tissue distribution [2]. They are abundant in various types of endocytic membranes, including exosome-generating MVBs [8], and have been widely used as exosomal markers because of their prevalence in exosomes and their role in exosome biogenesis, protein loading and sorting of cargoes into exosomes [64]. We used CD9 to mark exosomes, by fusing it with either Dendra-2 or mCherry to track the transmission of exosomes between interconnected hippocampal neurons. Cells were electroporated with each plasmid directly before plating and seeded separately into microfluidic chambers according to model 1 (red - > Ch1, green - > Ch2) (Fig. 1a).

**Fig. 2:**
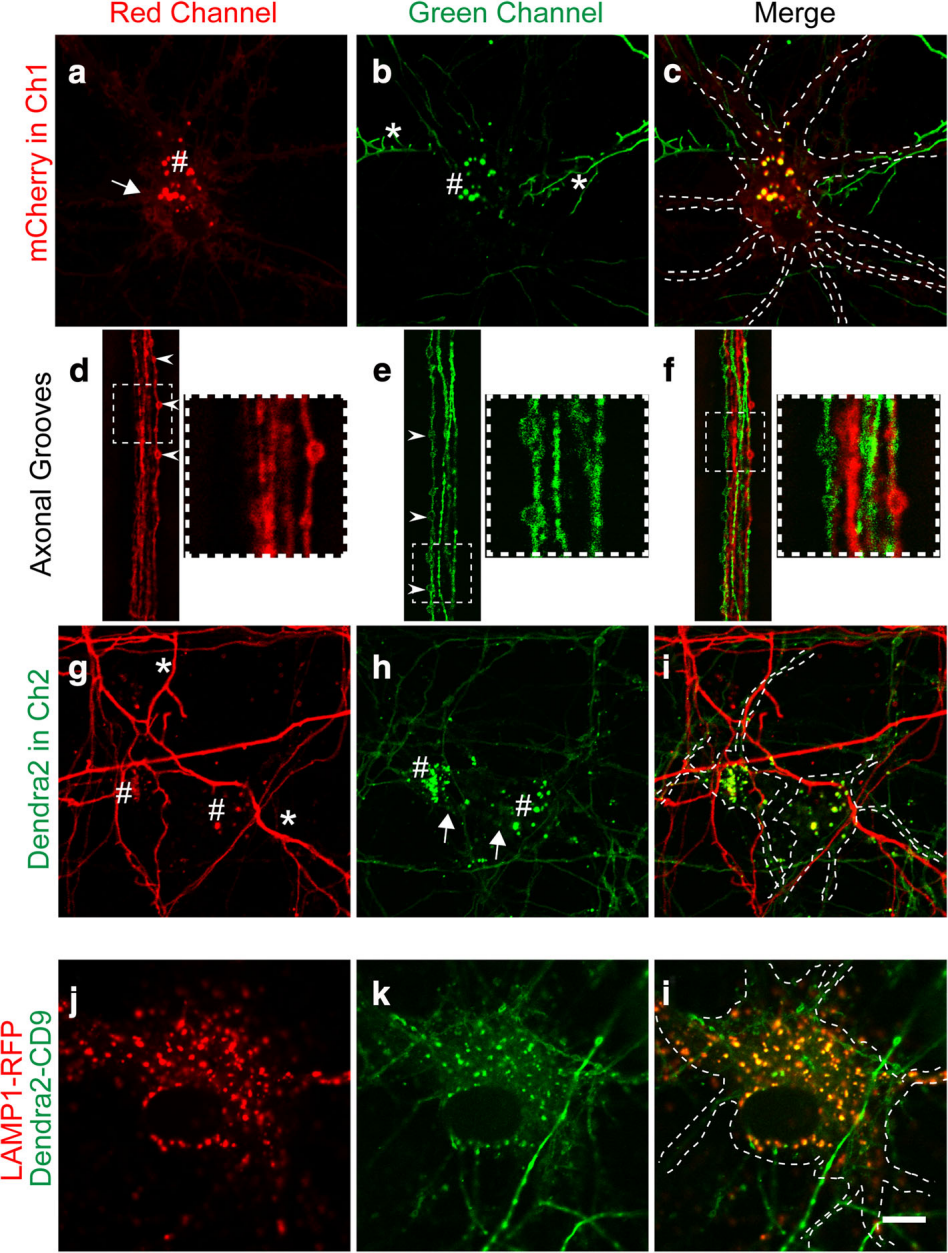
Transmission of exosomes between interconnected neurons. Culture performed according to Model 1, with neuron A being labeled with mCherry-CD9 (red) and neuron B labeled with Dendra2-CD9 (green). **a-c** Confocal images of mCherry-CD9-positive neurons in Ch1. The red channel (**a**) shows that CD9 is detected in the neuronal plasma membrane (**a**, white arrow) and is strongly present in endosomal somatic punctae (**a**, #). The green channel (**b**) reveals that Dendra2-positive axons (_*_) from Ch2 can reach mCherry-CD9-positive neurons in Ch1. Interestingly, red neurons only show green fluorescence in endosomal punctae when they are close to green axons (**b**, #). **d-f** Images of axonal grooves revealing that axons grow in both directions but never exchange fluorescence. White arrowheads indicate CD9-positive globular enlargements, which appear to be migrating endosomes based on single color fluorescence. The square (dashed lines) outlines a magnification of the axonal region (right square). **f** The magnification shows that the axonal endosomes are either green or red, but do not display both colors. This indicates that the fluorescence exchange found in the soma is post-synaptic. **g-i** Images of Ch2 containing Dendra2-CD9-expressing neurons. Similarly, Dendra2-expressing green endosomal punctae in Ch2 (**h**, #) show red fluorescence (**g**, #) when in proximity to red axons (**g**, _*_) projecting from Ch1. White arrows indicate the plasma membrane. **j-l** Colocalization of Dendra2-CD9-expressing endosomal punctae with the late endosome marker LAMP1 tagged with RFP (transduced with a baculovirus). Scale bar: 10 μm for all images

As expected, fluorescence was detected that delineated the plasma membrane of hippocampal neurons (Fig. 2a). In addition, somatic punctae representing endosomes were visualized that displayed a much stronger signal (Fig. 2a). Interestingly, the red endosomal punctae in the cell bodies in Ch1 also exhibited a green fluorescence signal in those neurons that were in close proximity to, and possibly interconnected to, Dendra2-CD9-expressing and -projecting axons (Fig. 2 b-c). Importantly, we observed that the exchange in fluorescence was restricted to the endosomal punctae, given that axons with different colors running in parallel in opposite directions within the microgrooves did not exhibit double fluorescence (Fig. 2 d-f). Similarly, we found no evidence for a fluorescence exchange at the plasma membranes. The reverse was also true, in that green cell bodies (Fig. 2 g-i, Ch2) acquired red fluorescence when they were in close proximity to mCherry-CD9-positive axons (Fig. 2i). For endosome quantification, we used segmentation of fluorescence confocal images and determined the percentage of endosomal punctae having both colors, which was 34.5% ± 5.8% (mean ± SEM, *n* = 6, 22 neurons), indicating a potential transfer of exosomes between neurons. Together, these results suggest a trans-synaptic transfer of exosomes that are generated by secretory endosomes. To confirm the endocytic origin of the visualized Dendra2-CD9-positive somatic punctae, we demonstrated colocalization with the late endosomal marker LAMP-1 (Fig. 2 j-l), a protein that remains on the limiting membrane of late endosomes during the formation of MVBs [49].

### A subset of exosomes are internalized and passed on to a third interconnected neuron

**Fig. 3:**
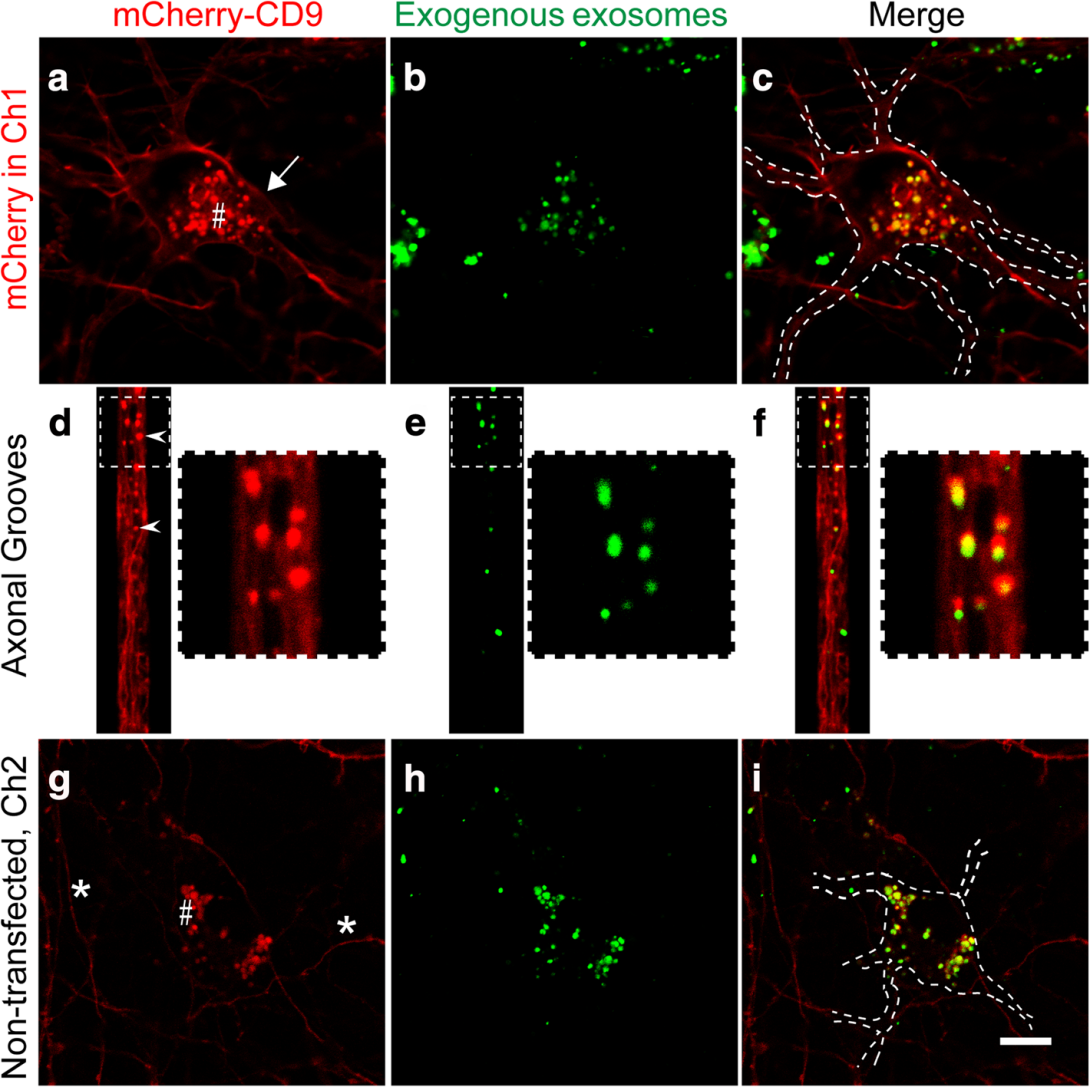
Neurons internalize exosomes but also share them with interconnected neurons. Culture performed according to Model 2, with neuron A-derived exosomes labeled with PKH67 (green), neuron B labeled with mCherry-CD9 (red) and neuron C being unlabeled (no color). **a-c** Confocal images in Ch1 containing mCherry-CD9-labeled neurons. The red channel (**a**) shows CD9 in the neuronal plasma membrane (**a**, white arrow) and a strong signal in endosomal somatic punctae (**a**, #). The green channel (**b**) reveals that PKH67-green is also detectable in somatic endosomal punctae where it colocalizes with endogenous somatic endosomes in red (**c**). **d-f** mCherry-CD9-labeled axons extending towards Ch2 and transporting red endosomal punctae (d, white arrowheads), which also carry green exogenous exosomes labeled with PKH67 (**e**-**f**). The square (dashed line) outlines a magnified axonal region. **f** Magnification showing that the axonal endosomes are labeled with both colors, meaning that they contain endogenous and exogenous cargoes. This implies that exogenous exosomes are axonally transported together with endogenous vesicles. **g-i** Hippocampal neurons in Ch2 that were not electroporated. These neurons only acquired red somatic endosomal punctae (**g**, #) when in proximity to red axons projecting from Ch1 (**g**, _*_). Endosomal punctae also show PKH67 green fluorescence (**h**-**i**), indicating post-synaptic acquisition of both exogenous and endogenous exosomes. Scale bar: 10 μm for all images

Having demonstrated the transfer of exosomes between two interconnected neurons, we sought to investigate whether all transferred exosomes find their final destination in the neurons that internalized them or whether some exosomes might have a somatic ‘free pass’ and be transferred to a third, interconnected neuron, thereby potentially increasing the pathogenic radius of action of exosomes. As outlined in Fig. 1b, a proxy for this situation is the treatment of neuron B in Ch1 with exosomes isolated from neuron A (rTg4510 brains) by labeling their membranes with the green fluorescent dye PKH67 (Fig. 1b, model 2). After internalization, exosomes would be processed neuron B, either by lysosomal degradation or by transfer of the exosomal content to the cytosol. We asked, however, whether a subset of the neuron B-internalized exogenous exosomes would be transported to neuron C in Ch2. To differentiate between the neurons in Ch1 and Ch2, only those in Ch1 were electroporated with mCherry-CD9 whereas those in Ch2 lacked fluorescence signal. As expected, PKH67-positive exogenous exosomes were internalized by the neurons in Ch1 and 67.0% ± 9.8% (mean ± SEM, *n* = 6, 41 neurons) of the endosomal particles contained both colors (Fig. 3 a-c). Interestingly, we also observed that the red projecting axons in the microgrooves contained migrating endosomes that were positive for both mCherry-CD9 and PKH67 (Fig. 3 d-f). Furthermore, neurons in Ch2 in close proximity to these red projecting axons exhibited somatic endosomal fluorescent punctae in which the two fluorescent signals colocalized (Fig. 3 g-i), with 40.3% ± 5.0% (mean ± SEM, n = 6, 18 neurons) of the endosomes containing both red and green fluorescence. In addition, an early pilot characterization of model 2 revealed that a subset of the exosomes isolated from rTg4510 mice added to Ch1 exhibited colocalization with human tau and, with only a fraction of the exosomes migrating into Ch2, an even smaller subset carried tau seeds as demonstrated with an antibody for human tau (Additional file 1: Figure S2). Together, these results support the notion that the endosomes containing internalized exogenous exosomes fuse with endogenous endosomes containing intraluminal nanovesicles, which are fated to be secreted as mCherry-CD9-positive exosomes, potentially contributing to the spreading of pathological human tau.

### Super-resolution microscopy supports fusion events between endosomes containing endogenous and exogenous exosomes

The results described in Fig. 3 revealed the presence of a number of fluorescent punctae moving along axons and presenting a signal from endogenous and exogenous membranes that we hypothesized to be derived from intraluminal nanovesicles (exosomes). However, the size of the punctae (0.5–1.5 μm approximately) visualized by confocal microscopy corresponded more with endosomes than exosomes (30–150 nm). We therefore turned to super-resolution microscopy in order to visualize discrete fluorescent particles inside the endosomes and better demonstrate the potential ‘hijacking’ of endogenous secretory endosomes.

**Fig. 4:**
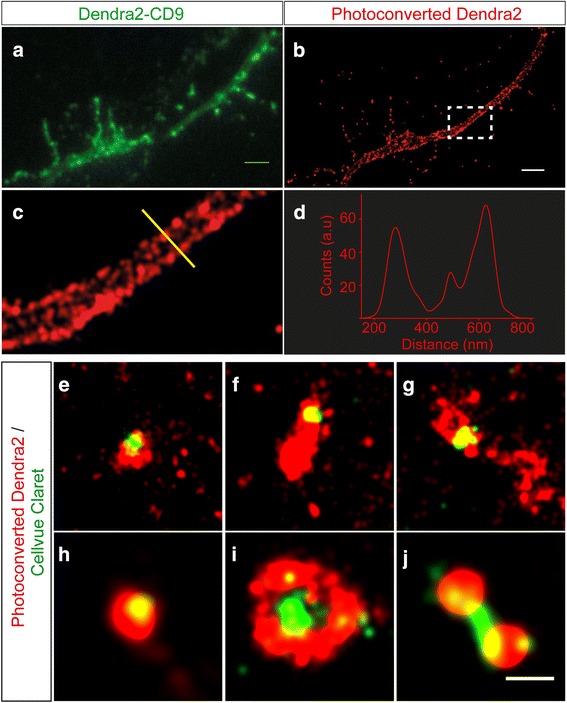
Super-resolution microscopy reveals that endosomes can carry both exogenous and endogenous exosomes. Culture performed according to Model 2, with neuron A-derived exosomes being labeled with the CellVue® Claret far-red fluorescent membrane dye (pseudocolored in green), neuron B labeled with Dendra2-CD9 (natively green but red when photoconverted) and neuron C containing no label (no color). **a** Conventional and (**b**) super-resolution images of a dendrite expressing Dendra2-labeled CD9. In the high-resolution image, structural features such as the plasma membrane become visible (scale bar 2 μm). **c** Magnification of the outlined rectangular region in (**b**). **d** Cross-section along the yellow line in (**c**) where the width of the dendrite and the thickness of the plasma membrane were measured. **e-j** Examples of colocalized endogenous endosomes and exogenous exosomes. **e**, **f** and **g**) showing events detected in axons. Panels h, i and j illustrate fusion events in soma and dendrites. In (**h**) and (**i**) exogenous exosomes are found close to the center of endosomal structures. Endosomal intraluminal nanovesicles cannot be resolved in red endosomal structures, but they contribute to the broader red fluorescence in the structure (ie: **f**, **h** and **i**). Scale bar 500 nm

We used stochastic super-resolution in which the fluorescence emission of a photoconvertible protein (Dendra2) or a synthetic organic fluorophore (CellVue Claret) can be sequentially activated in a semi-controlled manner to generate blinking of single fluorophores, thereby allowing for single molecule localization-based super-resolution imaging [9, 24, 53]. Using this technique, image resolution is achievable in the order of tens of nanometers, well below the diffraction limit of conventional microscopy. To analyze the endosomal punctae that contain colocalized endogenous and exogenous exosomes, we electroporated the neurons in Ch1 with Dendra2-CD9. In its native form Dendra2 is a fluorescent protein that emits green light. However, when illuminated at the low power level of 405 nm laser light, it is photoconverted to red fluorescence [31], and this ‘blinking’ generated by the color switch is used to determine the position of each individual fluorophore. This property allowed us to determine the localization of CD9 molecules in the plasma and endosomal membranes with a precision of 25 nm (Fig. 4 a-j).

Using the model 2 design (Fig. 1b), Dendra2-CD9-transfected neurons in Ch1 were treated with exosomes the membranes of which were labeled with CellVue Claret, a far-red dye with a chemical structure similar to that of other photo-switchable fluorescent probes [19, 59]. We used photoconversion of Dendra2 to locate endosomal membranes with a precision of 25 nm and the photo-switch of CellVue Claret to localize the membranes of exogenous exosomes with precision of 35 nm (Fig. 4 e-j).

Fig. 4a shows a representative conventional image of a dendrite the plasma membrane of which was labeled with Dendra2-CD9. Subsequent photoconversion and localization of single Dendra2 molecules produced a super-resolved image of the same dendrite (Fig. 4b). Previously obscured details including the plasma membrane of the dendrite can now be clearly resolved (Fig. 4b). Due to the limited localization precision of Dendra2, the neuronal plasma membrane appears as a 41 ± 4 nm thick dotted line as determined for the cross-section (yellow line in Fig. 4c, graphed in Fig. 4d). However, we were able to discriminate between the plasma membranes of axons and dendrites, and the endosomal particles located between them. Many endogenous endosomes in the neuronal soma, axons and dendrites in red colocalized with the exogenous exosomes labeled with CellVue Claret pseudocolored in green (Fig. 4 e-j). Fusion events between both types of endosomes were evident in endosomal particles located in axons (Fig. 4 e-g) as well as in the somata and dendrites (Fig. 4 h-j). This is consistent with the incorporation of exogenous exosomes into host neurons and their endosomes (green in Fig. 4 e-j). However, as our localization precision was only 35 nm, it did not allow us to visualize potential intraluminal nanovesicles as discrete particles, probably because these vesicles lie very close to one another inside endosomes. Instead, we observed an apparent uneven thickness of endosomal structures. For example, Fig. 4h and f shows the fusion between endogenous (red) and exogenous (green) particles. It is evident that the endogenous endosome is larger than the exogenously acquired exosome but potential internal nanovesicles (red) cannot be visualized.

### Electron microscopy reveals the hijacking of endogenous endosomes at a high resolution

**Fig. 5:**
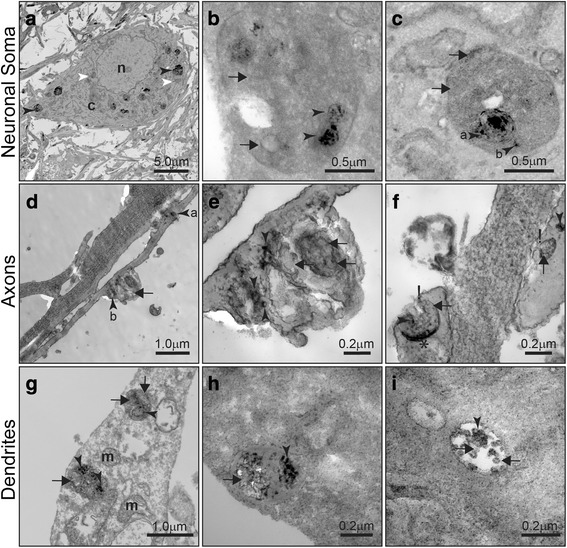
Hijacking of endogenous endosomes revealed by electron microscopy. Hippocampal neurons in microfluidic devices in Ch1 according to model 2 were treated with exosomes isolated from mouse brains and labeled with FM1–43FX, a fluorescent dye that reacts with diaminobenzidine (DAB) to form insoluble dark precipitates that are visualized by electron microscopy. **a** Electron microscopy image of a neuronal soma in Ch1, showing that the majority of endosomes contain DAB-positive exosomes of exogenous origin (black arrowheads). A few endosomes are DAB-negative (white arrowheads). **b** High magnification of endosomes located in the neuronal soma, showing a mixture of exogenous DAB-positive exosomes (black arrowheads) and endogenous DAB-negative intraluminal nanovesicles (black arrows). **c** Somatic endosome showing engulfment and fusion with a smaller endosome containing DAB-positive exosomes (black arrowhead “**a**”). An exogenous DAB-positive exosome can be seen close to the fused endosome (black arrowhead “**b**”). Endogenous DAB-negative intraluminal nanovesicles are also visualized (black arrows). **d-f** Endosomes found in axons. (d) Low magnification of axons transporting a small DAB-positive endosome (black arrowhead “**a**”) and a large endosome in front of it (black arrowhead “**b**”). **e** High magnification of a large axonal endosome containing a mixture of DAB-positive (black arrowheads) and DAB-negative vesicles (black arrows). **f** Axonal termini showing endosome fusion with the plasma membrane (indicated with “!”) during exosome release and potential residue of the back-fusion with the limiting membrane of the endosome (_*_). Exogenous DAB-positive (black arrowheads) and endogenous DAB-negative exosomes (black arrows). **g-i** Endosomes found in dendrites. **g** Low magnification of a dendrite demonstrating the presence of endosomes carrying both DAB-positive exosomes (black arrowheads) and DAB-negative intraluminal nanovesicles (black arrows). **h** and **i** High magnification of dendritic endosomes carrying DAB-positive exosomes less than 100 nm diameter (black arrowheads) together with DAB-negative vesicles of a similar size (black arrows). m, mitochondria; n, nucleus

Super-resolution images strongly support fusion events between endosomes containing either endogenous or exogenous intraluminal nanovesicles (exosomes). However, we were not able to visualize discrete intraluminal vesicles. Given that electron microscopy provides substantially higher resolution than super-resolution microscopy [25], we again adopted model 2 (Fig. 1b) and performed electron microscopy after first labeling the membranes of exogenous exosomes with FM1–43FX. When specimens are fixed, this fluorescent probe oxidizes the substrate diaminobenzidine (DAB), thereby converting it into a precipitated and electron-dense reaction product [46]. Photooxidized DAB can therefore be used to track vesicles by electron microscopy and differentiate between endogenous and exogenous vesicles [27, 33, 34, 37]. We found that the majority of endosomes in the neuronal soma contained exogenous material, as evident by the DAB precipitate (Fig. 5a, black arrowheads), and that only a few endosomes were DAB-negative, indicative of an exclusively endogenous origin (Fig. 5a, white arrowheads). This is also shown in the high magnification images that reveal a mixture of DAB-positive (black arrowheads) and DAB-negative (black arrows) intraluminal nanovesicles (Fig. 5 b-c). Fig 5c supports the notion that more than one fusion/internalization event occurred for the imaged endosomes (1.2 μm), as shown by the internalization of an endosome of approximately 400 nm containing smaller DAB-positive intraluminal nanovesicles (Fig. 5c, black arrowhead “a”). However, a DAB-positive intraluminal nanovesicle of less than 100 nm located outside “a” probably has been internalized during a separate fusion event (Fig. 5c, black arrowhead “b”).

Interestingly, when axons were imaged (Fig. 5 d-f), we were able to observe small endosomes containing DAB-positive nanovesicles moving freely inside the axonal lumen (Fig. 5d, black arrowhead “a”). However, we also noted a large convoluted endosome which appeared to stretch the axonal lumen and membrane (Fig. 5d, black arrowhead “b”). High magnification images of this axonal endosome revealed a complex structure with a mixture of endogenous and exogenous intraluminal nanovesicles (Fig. 5e). It is worth noting that because FM1–43FX exosomes were only added to the cell bodies in Ch1, the endosomes carrying the DAB-positive material (Fig. 5 d-f) were quite likely moving anterogradely. Furthermore, the axonal termini showed fusion events with the plasma membrane in what appeared to be the secretion of exosomes (Fig. 5f). Dendrites contained a similar type of ultrastructure, exhibiting endosomes containing a mixture of exogenous DAB-positive nanovesicles and endogenous DAB-negative vesicles (Fig. 5 g-i). Together, the cumulative evidence of super-resolution and electron microscopy data strongly supports the notion that endosomes containing endogenous intraluminal nanovesicles fuse with endosomes loaded with internalized exogenous exosomes. The fact that these mixed endosomes move in dendrites and axons and are presumably secreted, suggests that exogenous exosomes can hijack the secretory endosomal pathway of neurons and be secreted in conjunction with exosomes of endogenous origin.

## Discussion

Our study supports the notion that exosomes are invasive, hijacking the endosomal secretory machinery of the cells that internalized them to achieve a longer distance of action and a potentially higher pathogenicity. Previous studies have established that early endosomes arise from primary endocytic vesicles which fuse with each other to form a larger endocytic structure [40]. It is likely that endocytic vesicles containing internalized exosomes fuse with endocytic vesicles containing different cargoes and that, during endosome maturation towards the generation of intraluminal nanovesicles, this results in a late endosome sheltering both endogenous vesicles and exosomes taken up from another cell. It can be assumed that most of the internalized exosomes are used by the cell itself, by either undergoing lysosomal degradation or recovery of some components via the trans-Golgi network [39, 57], or by the release of exosomal contents into the cytosol via back-fusion mechanisms [10, 63]. However, our data show that not all internalized exosomes are destined for destruction or recovery, as some are actually re-secreted together with endogenous exosomes.

The hijacking of endosomes is not unique to exosomes. For instance, some enveloped RNA viruses hijack MVBs in order to be released from infected cells [17]. DNA viruses, such as the herpes virus, hijack the exosomal pathway of their host to facilitate virion assembly, promote the export of host proteins involved in immune regulation, and transfer viral-derived molecules that can assume activity in recipient cells [54]. Even the bacterium *Chlamydia trachomatis* has the capacity to hijack intracellular trafficking and lipid transport pathways of the host cell in order to promote infection [47]. However, different from the mechanism that occurs with intracellular pathogens, the hijacking of endosomes as revealed in our study does not require genome replication as with viruses or bacteria.

Perhaps the hijacking mechanism most similar to that which we report is used by the proteinaceous lethal toxin known as anthrax which exploits MVBs. Anthrax toxins can persist in intraluminal nanovesicles for days, fully sheltered from proteolytic degradation in MVBs and can be delivered to the extracellular medium as exosomes [1]. It is tempting to speculate that the fusion of exogenous exosomes with endosomes destined to secrete intraluminal nanovesicles would equally shelter the exogenous exosomes from proteolytic degradation in the MVBs which could eventually be secreted together with de novo generated exosomes. An alternative mechanism could be that, given the endocytic origin of exosomes, these contain markers for secretion and drive a subpopulation of exosomes into the secretory pathway. Comparison with the internalization of microvesicles, which are not of endocytic origin [38, 45], might provide insight into this possibility.

Tau protein aggregation is a hallmark of AD and other neurodegenerative diseases collectively termed tauopathies. Tau accumulation in the brain hinders neuronal physiology at many levels, including axonal transport and synaptic transmission, mitochondrial and proteasomal functions, induction of endoplasmic reticulum stress and even nuclear effects on chromatin relaxation [28, 50]. It is assumed that the ability of exosomes to carry misfolded or aggregated proteins significantly enhances the progression of tauopathies in a manner similar to what has been reported for prions [6, 38, 50, 51]. However, it has been proposed that an increase in pathogenic exosomes could end up in traffic jams during endosome transport, which could cause a reduction of glutamate receptor recycling [60]. Traffic jams could increase the number of intraluminal nanovesicles in the MVBs, thereby increasing the number of released exosomes; or increase the content of exosomes with AD-associated proteins like tau or Aβ, leading to an accelerated spread of disease [60]. We observed that brain-derived exosomes are strongly internalized by neurons, resulting in a somata with high numbers of endosomes. Similarly, axons exhibited endosomes of varied sizes moving inside the axonal lumen, where sometimes massive endosomes were caught stretching the axonal membrane. We reasoned that huge endosomes, probably generated by the upregulated endosomal activity, are more difficult to transport along axons and might end up in traffic jams that could strongly affect neuronal physiology. Interestingly, synaptic activity increases the secretion of exosomes [18, 43], and hippocampal hyperactivity has been observed in patients with mild cognitive impairment [7], where the compounded action of both mechanisms might also generate endosomal traffic jams acting as upstream drivers of AD pathogenesis.

In this study, we demonstrated features of exosome spreading between interconnected neurons in agreement with what is expected of this type of vesicle of endocytic origin. However, we also provide evidence for a novel hijacking mechanism of endosomes by exogenous exosomes, which might result in a longer-distance action and therefore increase the pathogenic potential and the radius of action of the exosomes. These intriguing findings demonstrate that exosomes are more invasive that previously anticipated acting as amplifiers in the spread of pathogenic molecules in neurodegenerative diseases.

## Conclusion

Our study reveals an unusual intracellular trafficking of exosomes in that not all internalized exosomes are degraded or their constituents recovered by the cell. Instead, endosome hijacking leads to the generation of a persistent subpopulation of exosomes with a longer distance of action and potential pathogenicity.

## Additional file

Additional file 1: Supplementary information. **Figure S1.** Abandoned model establishing neuronal circuits using triple chamber microfluidics devices. **Figure S2.** Internalized and migrating exosomes show human tau. (DOCX 2897 kb)

## Abbreviations

Aβ: Amyloid-β
AD: Alzheimer disease
APP: Amyloid precursor protein
DAB: Diaminobenzidine
DIV: Days in vitro
E: Embryonic day
MVBs: Multivesicular bodies
NFTs: Neurofibrillary tangles
