# Supplementary material for: Exosomes taken up by neurons hijack the endosomal pathway to spread to interconnected neurons
Source: Acta Neuropathol Commun. 2018 Feb 15;6:10. doi: 10.1186/s40478-018-0514-4 (PMC5815204; doi:10.1186/s40478-018-0514-4)
Supplement: Supplementary file 1 — Supplementary information. Figure S1. Abandoned model establishing neuronal circuits using triple chamber microfluidics devices. Figure S2. Internalized and migrating exosomes show human tau. (DOCX 2897 kb) [file 40478_2018_514_MOESM1_ESM.docx]

Supplementary information for:

**Exosomes taken up by neurons hijack the endosomal pathway to spread to interconnected neurons**

Juan Carlos Polanco^1^, Chuanzhou Li^1^, Nela Durisic^2^, Robert Sullivan^2^ and Jürgen Götz^1, *^

1. *Clem Jones Centre for Ageing Dementia Research (CJCADR), Queensland Brain Institute (QBI), The University of Queensland, Brisbane QLD 4072, Australia.*
2. *Queensland Brain Institute (QBI), The University of Queensland, Brisbane QLD 4072, Australia.*


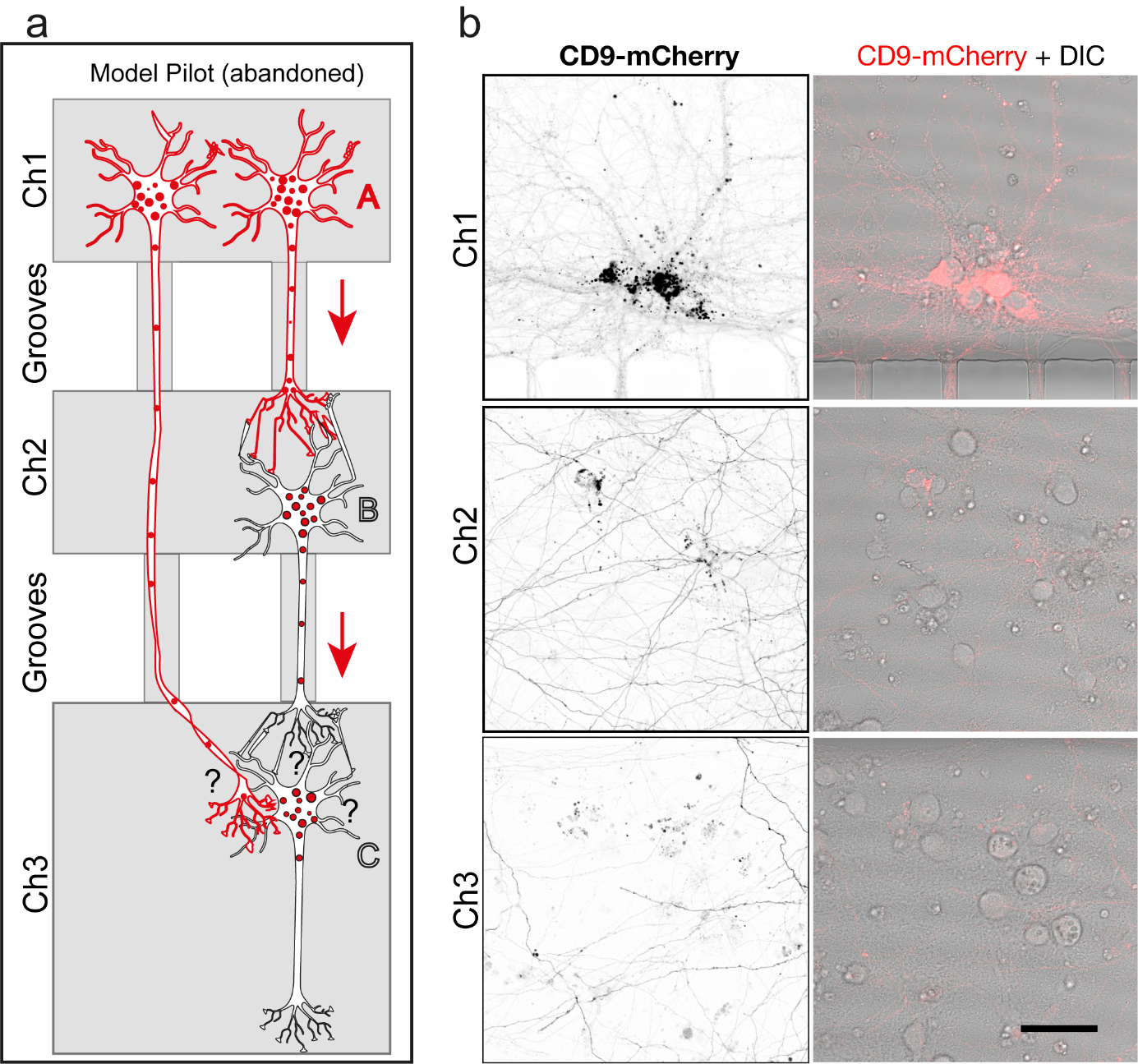


**Fig. S1 Abandoned model establishing neuronal circuits using triple chamber microfluidics devices.** (**a**) Sizes of neurons and culture chambers are not shown to scale. Neurons expressing the tetraspanin CD9 together with a red fluorescent tag were used to track exosomal, endosomal and plasma membranes. Pilot model with triple chamber microfluidics devices (Ch1, Ch2, Ch3) interconnected with microgrooves for axonal projection. The circuit of three interconnected neurons (A-B-C) is corrupted by the axonal connection A-C. As shown for DIV8-9 cultures, red axons from Ch1 reach Ch3 irrespective of whether cells are present in Ch2 or not. Therefore, it is not possible to determine whether the fluorescence in C is originating from A or indirectly by passing first through B. This model was therefore abandoned. (**b**) Culture performed with neuron A electroporated with mCherry-CD9 and seeded in Ch1 only, with neuron B (Ch2) and neuron C (Ch3) being unlabeled (no color), revealing axonal projection of red axons from Ch1 to Ch3. Scale bar: 50 µm.


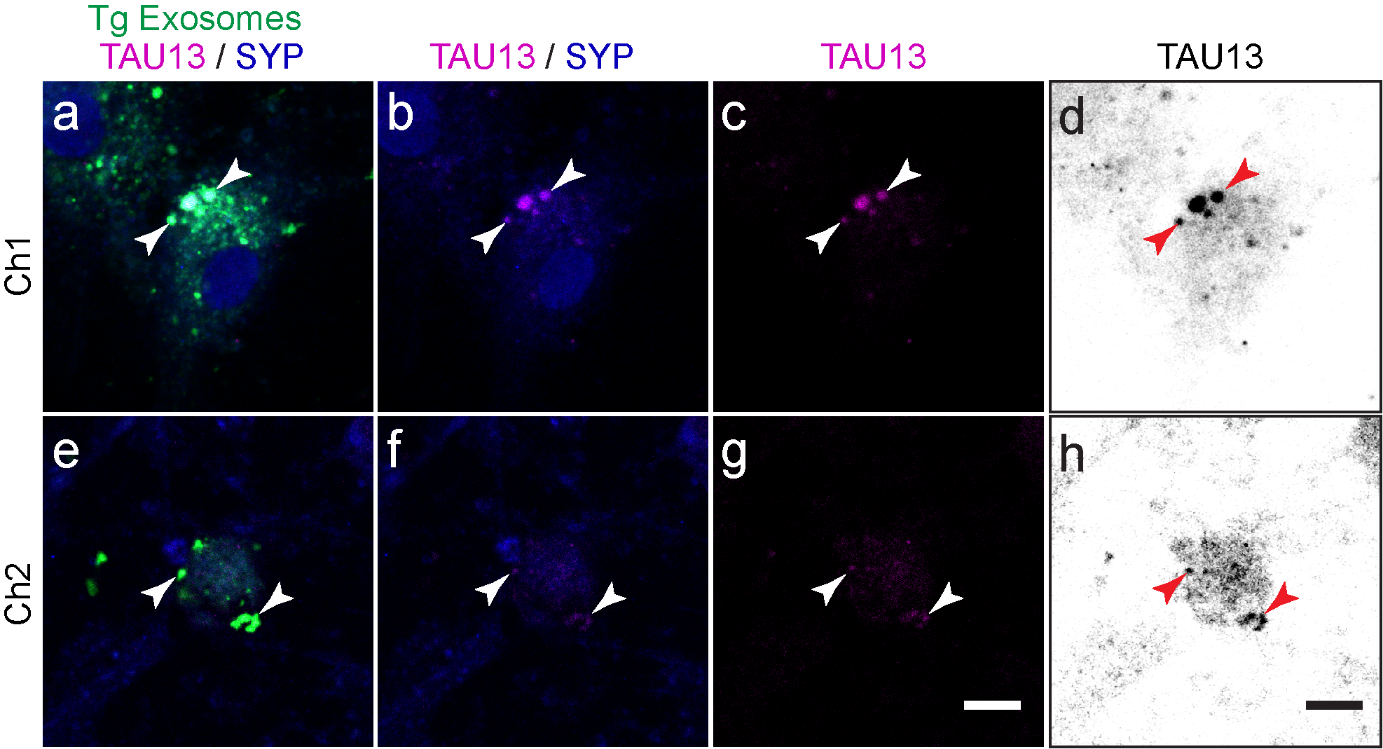


**Fig S2 Internalized and migrating exosomes show human tau.** Culture performed according to model 2, with neuron A-derived exosomes labeled with FM1-43FX (green), and neurons B and neuron C being unlabeled (no color). (**a-d**) Confocal images in Ch1 containing hippocampal neurons. The far-red channel shows human tau detected with antibody TAU-13, the green channel shows rTg4510-derived exosomes (Tg) labeled with FM1-43FX (green) and the blue channel detects synaptophysin (SYP). A stronger signal for human tau is found in Ch1 where the majority of internalized exosomes remain (c, d). Arrowheads indicate internalized exosomes carrying human tau. (**e-h**) A lower signal is detected in Ch2 (g, h). Visualization is facilitated by imaging the detected florescence in gray scale with inverted black to white values (d, h). Scale bars: 10 µm.
